# Effect of hosts on competition among clones and evidence of differential selection between pathogenic and saprophytic phases in experimental populations of the wheat pathogen *Phaeosphaeria nodorum*

**DOI:** 10.1186/1471-2148-11-188

**Published:** 2011-07-01

**Authors:** Rubik J Sommerhalder, Bruce A McDonald, Fabio Mascher, Jiasui Zhan

**Affiliations:** 1 Institute of Integrative Biology, ETH Zurich, LFW, Universitaetstrasse 2, Zürich, CH-8092, Switzerland; 2 Agroscope Changins-Wädenswil Research Station ACW, P.O. Box 1012, Nyon, CH-1260 Switzerland; 3 Key Lab for Biopesticide and Chemical Biology, Ministry of Education, Fujian Agriculture and Forestry University, Fuzhou, 350002, China; 4 Fujian Key Lab of Plant Virology, Institute of Plant Virology, Fujian Agriculture and Forestry University, Fuzhou, 35002, China

**Keywords:** host selection, experimental evolution, microsatellite, *Stagonospora nodorum*, cultivar mixture, genetic diversity

## Abstract

**Background:**

Monoculture, multi-cropping and wider use of highly resistant cultivars have been proposed as mechanisms to explain the elevated rate of evolution of plant pathogens in agricultural ecosystems. We used a mark-release-recapture experiment with the wheat pathogen *Phaeosphaeria nodorum *to evaluate the impact of two of these mechanisms on the evolution of a pathogen population. Nine *P. nodorum *isolates marked with ten microsatellite markers and one minisatellite were released onto five replicated host populations to initiate epidemics of Stagonospora nodorum leaf blotch. The experiment was carried out over two consecutive host growing seasons and two pathogen collections were made during each season.

**Results:**

A total of 637 pathogen isolates matching the marked inoculants were recovered from inoculated plots over two years. Genetic diversity in the host populations affected the evolution of the corresponding *P. nodorum *populations. In the cultivar mixture the relative frequencies of inoculants did not change over the course of the experiment and the pathogen exhibited a low variation in selection coefficients.

**Conclusions:**

Our results support the hypothesis that increasing genetic heterogeneity in host populations may retard the rate of evolution in associated pathogen populations. Our experiment also provides indirect evidence of fitness costs associated with host specialization in *P. nodorum *as indicated by differential selection during the pathogenic and saprophytic phases.

## Background

The evolution of pathogens is widely believed to be one of the major challenges facing agriculture and medicine [1,2]. Experimental studies focused on the evolution of pathogens, including the emergence of virulence and pathogen adaptation to changing agricultural and medical practices, can provide critical information for more effective management of infectious diseases. In medicine, infectious diseases are mitigated mainly through the application of antimicrobial substances such as antibiotics. While pesticides such as fungicides are widely utilized in agricultural ecosystems, host resistance imposes many fewer environmental costs and is a more cost efficient approach to control plant diseases. In both agriculture and medicine, the efficacy of host resistance and antimicrobials usually decays over time as a result of the continuous evolution and adaptation of pathogens.

Plant pathogens are thought to evolve faster in agricultural ecosystems than in natural ecosystems [3-6]. Wild relatives are the primary sources of host resistance bred into cultivated crops. The disease resistance genes carried by these wild relatives of modern crop plants have coexisted with their pathogens for many thousands or millions of years in natural ecosystems. However, when these resistance genes are introgressed into modern crops and deployed in agricultural ecosystems, their value in controlling infectious diseases usually does not last for more than 10 years [7]. Temporal analysis of population dynamics also is consistent with the hypothesis of rapid pathogen evolution in agricultural ecosystems. For example US-1 was the predominant genotype in *Phytophthora infestans *populations around the world until the 1990s [8] but this genotype is rarely recovered since 2000. In the UK, a single *P. infestans *genotype called Blue-13-A2 was first detected in southern England in 2003 at a very low frequency. By 2007, this genotype was detected in all populations sampled across the UK and accounted for more than half of 1000 isolates assayed (J. Zhan & D. Cooke, unpublished data). These *P. infestans *examples illustrate how pathogen populations can experience rapid turnover as new genotypes with greater fitness emerge, spread, out-compete and replace earlier genotypes.

The evolution of pathogens can be influenced by the type of resistance, the amount of diversity found in host populations and the type of cropping system [9-12]. Modern agriculture is dominated by species monocultures grown at a high density. In these agricultural ecosystems, it is common for a single host cultivar or genotype carrying a major resistance gene to be grown over a large area. The limited genetic diversity in the host populations coupled with intensive use of major resistance genes can lead to rapid shifts in associated pathogen populations. A mutant with higher fitness that emerges in a pathogen population as a result of a single mutation event can quickly increase in frequency through strong directional selection and spread across entire fields or regions through natural or human-mediated migration.

Multi-cropping, where the same annual crop is grown in the same field more than once during the same year, is another common practice in modern agriculture, especially in countries experiencing a shortage of arable land. This practice may further accelerate the evolution of plant pathogens because locally adapted pathogen genotypes with a high parasitic fitness can steadily increase in frequency due to the year-around availability of the living host (i.e. a "green bridge" allows the parasitic phase of the pathogen life cycle to occur continuously).

It is hypothesized that the evolution of plant pathogens in agricultural ecosystems can be retarded by increasing genetic diversity of the host populations, by using partial resistance encoded by several genes and by avoiding multi-cropping systems. Increasing genetic diversity in host populations by mixing plants carrying different major resistance genes (e.g. cultivar mixtures or multilines) is thought to be an ecologically and evolutionarily sound approach to control plant diseases, particularly for airborne pathogens of cereals [13]. Increasing host diversity by using cultivar mixtures will impose disruptive selection on pathogen populations, i.e. pathotypes that are favored on one host will have lower fitness on the other hosts in the mixture [14-16], impeding their ability to evolve towards higher virulence, here defined as the damage a pathogen causes to its host [17]. On the other hand, because many fungal pathogens have large effective population sizes [18] and exhibit a mixture of sexual and asexual reproduction [19,20], they can rapidly obtain new pathogenicity factors through mutation or new combinations of pathogenicity factors through recombination and then maintain the novel combinations of pathogenicity factors through asexual reproduction. Thus extensive use of cultivar mixtures could lead to the development of complex races [21,22] that would be able to infect a large number of host genotypes carrying different major resistance genes.

Though less efficient, partial resistance is thought to offer a more durable method to control plant diseases than major-gene resistance because it works against all pathogen strains and selects equally against all pathotypes [23,24]. Partial resistance mediated by multiple genes is generally inherited as a quantitative trait [16,25,26], where each gene makes a minor but additive contribution to the overall resistance [27]. But selection can increase the frequencies of genes encoding higher virulence in pathogen populations infecting partially resistant hosts and reduce the effectiveness of quantitative resistance [9,19,28-31] though possibly at a slower pace compared to major resistance genes [32].

In contrast to multi-cropping, in single cropping systems an annual crop is grown for only 6-9 months of the year or different crops are rotated annually, forcing pathogens to undergo a saprophytic phase in their life cycle in which different strains not only compete with each other but also with other microbial species for nutrients and habitats. Pathogen genotypes that have a high parasitic fitness on living hosts may have a low saprophytic fitness on the dead host biomass. This trade-off could delay the emergence of highly parasitic pathogen strains in agricultural ecosystems characterized by single cropping and regular crop rotations.

**Figure 1:**
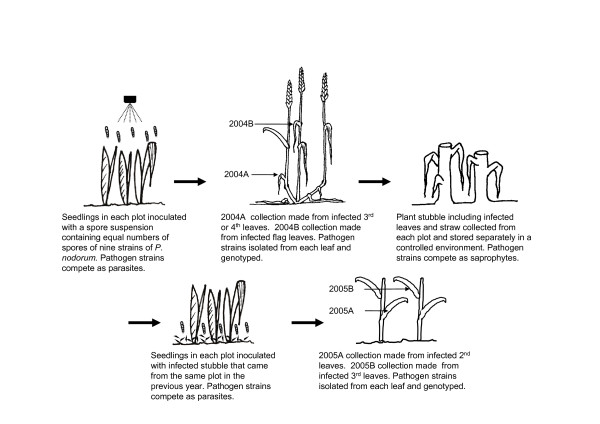
**Schematic of experimental design illustrating inoculation and sampling procedures used over the course of the 2-year field experiment conducted in Switzerland during the 2003-2004 and 2004-2005 winter wheat growing seasons**.

Much of our knowledge of pathogen evolution in agricultural ecosystems is drawn from theory or through historical inference from population surveys [33-35]. Experimental tests of pathogen evolution are limited and usually are conducted in controlled environments under laboratory or greenhouse conditions. Here we describe a test of pathogen evolution in a replicated experiment using sensitive molecular markers that could differentiate among pathogen isolates released into an unregulated field setting. This experimental evolution approach based on a mark-release-recapture strategy has now been successfully applied to understand the evolution of cereal pathogens including *Mycosphaerella graminicola *[20,32,36,37], *Rhynchosporium secalis *[37] and *Phaeosphaeria nodorum *[38]. In this study, we used this approach to investigate the evolution of the wheat pathogen *Phaeosphaeria nodorum*. The experiment was conducted over two years using five replicated host populations differing in levels of resistance and diversity. In the first year, the pathogen populations were introduced into each host population by artificial inoculation of the hosts with nine *P. nodorum *strains tagged with molecular genetic markers and mixed in equal proportions. In the second year, the pathogen populations were established using the infected straw and plant debris saved from the first year's experiment. During the experiment, two fungal collections were made in each of the two years. The recovered pathogen populations were assayed for their molecular markers so that frequencies of the inoculated isolates could be compared across hosts and sampling times (see Figure 1). With this experimental design, we were able to determine the effects of host diversity and resistance on the evolution of corresponding pathogen populations. The experimental design also allowed us to detect selection operating during both parasitic and saprophytic phases of the pathogen life cycle. The specific objectives of this experiment were to: i) infer the rate of pathogen evolution in an agricultural system; ii) determine the effect of host resistance on competition among genotypes in *P. nodorum *populations; iii) determine the effect of cultivar mixtures on clonal competition in *P. nodorum *populations; and iv) compare selection during the parasitic and saprophytic phases of the *P. nodorum *life cycle. Our previous data analyses indicated that isolates recovered from the experiment included the asexual progeny of the inoculated genotypes, airborne immigrants from outside of the experimental plots and recombinants arising from crosses between the inoculants and/or immigrants [39]. The results presented here consider only the effects of host selection and clonal competition among the asexual progeny of the inoculated genotypes. Evolutionary changes in the pathogen populations attributed to recombination and immigration were considered in a separate publication [39].

**Figure 2:**
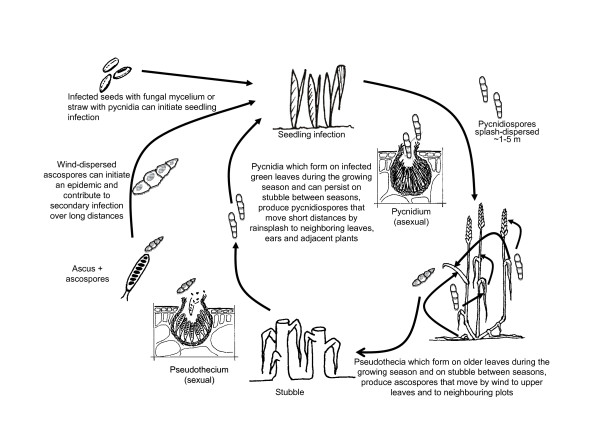
**Life cycle of *Phaeosphaeria nodorum *causing Stagonospora nodorum blotch of wheat**.

The heterothallic loculoascomycete *Phaeosphaeria nodorum *(Berk.) Castellani and Germano (syn. *Septoria nodorum *Berk.), the teleomorph form of *Stagonospora nodorum *(E. Müller) Hedjaroude (syn. *Leptosphaeria nodorum *E. Muller), causes Stagonospora nodorum leaf and glume blotch on wheat (*Triticum aestivum *L.). The pathogen can undergo both sexual and asexual reproduction (see Figure 2 for the life cycle) and has the ability to infect all above-ground plant parts during the parasitic phase [40-43]. The pathogen overwinters during its saprophytic phase on infected stubble [44] and can survive for several months [45] on wheat straw until the parasitic phase of the disease cycle is re-initiated. The primary inoculum includes infected seeds as well as pycnidiospores and ascospores. Asexual pycnidiospores are dispersed over short distances by rain-splash while sexual ascospores are wind-dispersed, therefore having the potential for long distance movement [46-49]. Ascospore-producing perithecia of *P. nodorum *can be formed during the host-free period in infested stubble on the soil surface [50,51] and during the growing season on infected plants [39].

## Results

### Recovery of inoculants

A total of 637 isolates matching the multilocus haplotypes of the inoculants were recovered from the inoculated plots. In addition to the inoculants, a large number of isolates (550) sampled over the course of this experiment were novel isolates with multilocus haplotypes that did not match the nine inoculants. The frequency of these novel haplotypes increased steadily over the course of the experiment. The majority of the novel genotypes were detected only once and the most frequent one was detected five times from two adjacent plots in 2005B.

### Variation in genotype frequencies among inoculants

**Figure 3:**
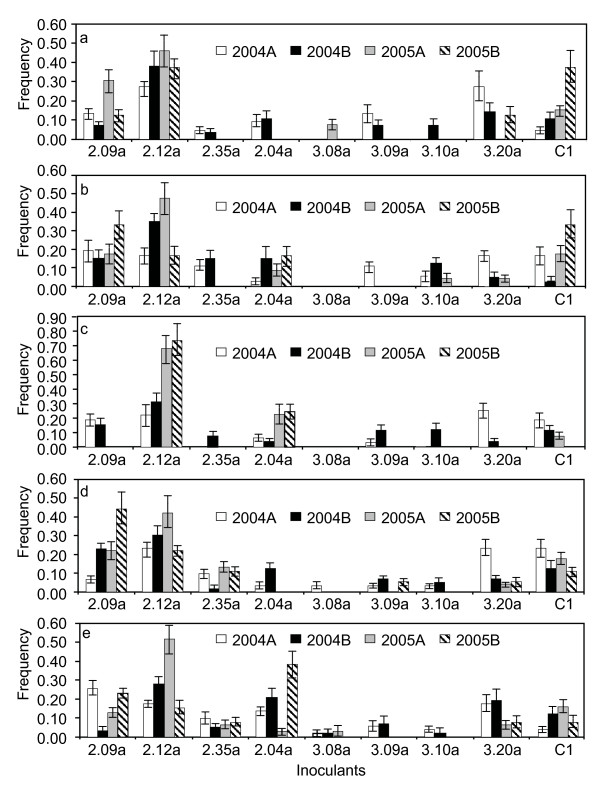
**Frequencies and their 95% confidence intervals of nine *Phaeosphaeria nodorum *isolates recovered from different host treatments during the 2004 and 2005 growing seasons**. Only the last part of each isolate name (See Table 1 for full names) is shown in the figure. A) Levis; B) Runal; C) 1:1 Mixture of Runal and Tamaro; D) Tamaro and E) Tirone.

Contingency χ^2 ^tests indicated that there were highly significant (P < 0.001) differences in genotype frequencies among the nine inoculants in the pathogen collections sampled from the different host treatments. Isolate SN99CH2.12a became well established and increased in frequency from 2004A to 2005A on all host treatments but decreased in frequency from 2005A to 2005B on four of five hosts (Figure 3). Isolate SN99CH2.09a established well at the beginning of the 2004 season on all host treatments. Its frequency increased from less than 20% in 2004A to about 35% in 2005B on the partially resistant cultivar Runal and increased from about 10% in 2004A to nearly 45% in 2005B on the partially resistant cultivar Tamaro. Isolate C1 established well on all host treatments. Its frequency steadily increased from less than 5% in 2004A to nearly 40% in 2005B on cultivar Levis but gradually decreased from 15% in 2004A to 0% in 2005B on the mixture. The frequency of isolate SN99CH2.04 increased on all host treatments except the cultivar mixture during the growing seasons (from 2004A to 2004B and from 2005A to 2005B) but decreased in frequency during the saprophytic phase (i.e. between 2004B to 2005A, Figure 3).

### Comparisons of genotype frequency among *P. nodorum *populations from different hosts

No significant differences in genotype frequency were detected between the pathogen populations collected from any pair of host treatments in 2004A (Table 1). But significant differences (pre-Bonferroni correction) were detected between two pairs of pathogen populations each in 2004B (Tirone-Runal and Tirone-Tamaro, Table 1) and 2005A (Levis-Tirone and Levis-Runal, Table 2). Pair-wise comparison was not conducted for the 2005B collection because of the small sample size. Following a Bonferroni correction for multiple comparisons, the hypothesis of no difference in population genetic structure among *P. nodorum *populations sampled from different hosts was rejected for the 2004B and 2005A collections. When all populations from the same time point were considered simultaneously using a multi-population comparison, significant differences in genotype frequencies were detected in collections 2004B, 2005A and 2005B but not in collection 2004A (Table 3). There was a clear pattern of increasing differences in genotype frequencies among pathogen populations sampled from different hosts over time (Table 3).

**Table 1:** Pair-wise comparisons for differences in genotype frequencies between *Phaeosphaeria nodorum *collections made from different hosts in 2004 based on a contingency χ^2 ^test.

	Levis	Runal	Mixture	Tamaro	Tirone
Levis	---	10.24 (7)	7.43 (6)	14.06 (8)	9.07 (8)
Runal	12.66 (7)	---	5.53 (7)	7.38 (8)	8.41 (8)
Mixture	5.42 (7)	9.29 (7)	---	6.69 (8)	11.37 (8)
Tamaro	7.32 (7)	13.39 (7)	4.86 (7)	---	14.98 (8)
Tirone	7.68 (8)	20.51 (7)**	14.59 (8)	16.33 (8)*	---

Above the diagonal are comparisons between populations sampled in early 2004 (2004A) and below the diagonal are comparisons between populations made in late 2004 (2004B). Values in bold font are significant after Bonferroni correction at a global alpha of 0.05 (p-value of 0.05/10 = 0.005) and values in parenthesis are degrees of freedom.

* Significant at p = 0.05 before Bonferroni correction

** Significant at p = 0.01 before Bonferroni correction

**Table 2:** Pair-wise comparisons for differences in genotype frequencies between *Phaeosphaeria nodorum *collections made from different hosts in 2005A based on a contingency χ^2 ^test.

	Runal	Mixture	Tamaro	Tirone
Levis	42.65 (8)****	10.81 (6)	5.64 (4)	12.03 (5)*
Runal		1.92 (5)	8.32 (6)	3.98 (7)
Mixture			7.8 (5)	3.9 (6)
Tamaro				4.76 (6)

Values in bold font are significant after Bonferroni correction at a global alpha of 0.0001 (p-value of 0.0001/10 = 0.00001) and values in parenthesis are degrees of freedom.

* Significant at p = 0.05 before Bonferroni correction

**** Significant at p < 0.0001 before Bonferroni correction

**Table 3:** χ^2 ^tests for difference in genotype frequencies among *Phaeosphaeria nodorum *populations sampled from different hosts at the same point in time.

Collection	**χ**^**2**^
2004A	37.99 (32)
2004B	46.12 (32)*
2005A	55.48 (32) **
2005B	51.67 (28) **

Values in bold font are significant after Bonferroni correction at a global alpha of 0.05 (p-value of 0.05/4 = 0.0125) and values in parenthesis are degrees of freedom.

* Significant at p = 0.05 before Bonferroni correction

** Significant at p = 0.01 before Bonferroni correction

### Changes in genotype frequencies among *P. nodorum *populations over time

Between 2004A and 2004B, significant changes in genotype frequencies prior to Bonferroni correction were observed in *P. nodorum *populations collected from Runal, Tamaro, and Tirone but not from Levis (Table 4). Significant changes prior to Bonferroni correction also occurred in the populations collected from Tamaro, Tirone and Levis between 2004B and 2005A (i.e. during the saprophytic phase) as well as between 2004A and 2005A. The changes in genotype frequencies were not significant in the populations sampled from the host mixture in any pair-wise comparisons over the two-year experiment. Significant differences in genotype frequency were detected in pathogen populations sampled from all host populations except the mixture when pathogen populations from three collections of the same host were considered together in a multi-population comparison (Table 4 Column 5). When data from different host treatments at the same sampling time were pooled to form a single population, all comparisons in genotype frequencies were significant both before and after Bonferroni correction (Table 5).

**Table 4:** Contingency χ^2 ^tests for differences in genotype frequencies between *Phaeosphaeria nodorum *collections made from the same host treatment at different sampling times.

	Pair-wise comparisons			Multi-population comparisons
	
	2004A vs. 2004B	2004B vs. 2005A	2004A vs. 2005A	
Levis	10.16 (7)	25.02 (8)***	17.50 (7) **	32.37 (16) **
Runal	16.28 (7)*	10.82 (6)	11.82 (7)	24.95 (14)*
Mixture	10.87 (7)	10.97 (8)	10.38 (6)	18.26 (16)
Tamaro	17.80 (8)*	17.25 (7)**	15.87 (8) *	30. 13 (16)*
Tirone	15.80 (8)*	15.60 (8)*	19.54 (8) **	31.64 (16) **

Values in bold font are significant after Bonferroni correction at a global alpha of 0.05 (p-value of 0.05/15 = 0.0032) and values in parenthesis are degrees of freedom. A Bonferroni correction was not applied for the multi-population analysis.

* Significant at p = 0.05 before Bonferroni correction

** Significant at p = 0.01 before Bonferroni correction

*** Significant at p = 0.001 before Bonferroni correction

**Table 5:** Contingency χ^2 ^tests for differences in genotype frequencies between *Phaeosphaeria nodorum *collections sampled at different times.

	2004B	2005A	2005B
**2004A**	22.63 (8)**	24.00 (8)**	34.83 (8)****
**2004B**	-	22.98 (8)**	44.67 (8) ****
**2005A**		-	40.57 (8) ****

Values in bold font are significant after Bonferroni correction at a global alpha of 0.05 (p-value of 0.05/6 = 0.0083) and values in parenthesis are degrees of freedom.

** Significant at p = 0.01 before Bonferroni correction

**** Significant at p < 0.0001 before Bonferroni correction

### Selection coefficients

Significant differences in selection coefficients were found on all treatments except the cultivar mixture. The average selection coefficients of the five most common isolates ranged from 0.03 to 0.83 across cultivars (Table 6). Isolates differed in their degree of adaptation to the different cultivars as indicated by the significant cultivar-by-isolate interaction in the analysis of variance for selection coefficients (Table 7). For example, isolate SN99CH2.04a displayed the highest fitness on cultivar Runal and SN99CH2.09a displayed the highest fitness on cultivar Tamaro. SN99CH3.20a, which was the only inoculated isolate carrying the *ToxA *gene, had selection coefficients ranging from 0.29 on Tirone to 0.80 on Runal. All isolates exhibited similar fitness on the mixture as indicated by no significant difference in their selection coefficients.

**Table 6:** Average selection coefficients and their standard deviations (in parentheses) for five released pathogen strains on each host treatment.

	Levis	Runal	Mixture	Tamaro	Tirone
SN99CH2.04a	0.51 (0.08) a	0.03 (0.03) a	0.34 (0.13) a	0.15 (0.14) c	0.42 (0.09) b
SN99CH2.09a	0.48 (0.10) a	0.59 (0.09) b	0.44 (0.15) a	0.03 (0.03) c	0.82 (0.07) a
SN99CH2.12a	0.27 (0.10) b	0.37 (0.11) c	0.43 (0.15) a	0.52 (0.05) b	0.32 (0.10) c
SN99CH3.20a	0.30 (0.11) b	0.80 (0.06) a	0.40 (0.16) a	0.71 (0.28) a	0.29 (0.09) c
C1	0.29 (0.12) b	0.83 (0.05) a	0.43 (0.14) a	0.67 (0.06) a	0.05 (0.04) b

Different letters following mean values in the same column indicate that selection coefficients differ significantly at *P *= 0.05.

**Table 7:** Analysis of variance for selection coefficients of the five most frequent inoculated strains of *Phaeosphaeria nodorum*.

Source	df	F-ratio	P value
Cultivar	4	15.56	0.0001
Isolate	4	130.28	0.0001
Cultivar * Isolate	12	145.87	0.0001

## Discussion

### Rapid change in the composition of *P. nodorum *populations

Because the epidemics were initiated by artificially inoculating the five host treatments with the same *P. nodorum *population (i.e. the mixture of nine marked strains in equal proportions), our null hypotheses were that the frequencies of the nine released isolates would be nearly equal in different host populations and that the genetic composition of these populations would not change over time. Instead, we observed significant differences in the frequencies of the released isolates and the majority of *P. nodorum *populations sampled from the five host treatments changed significantly over time. The differences in genotype frequency among the inoculated strains within a host population and among host populations sampled from different points in time could be due to random genetic drift or natural selection, but we believe that the observed differences in this case should be attributed mainly to selection. We have two lines of evidence supporting this hypothesis. 1) If genetic drift was the main factor, we would expect random changes in genotype frequencies among the *P. nodorum *populations sampled from different hosts. Instead, we found that temporal dynamics of the *P. nodorum *populations was strongly affected by the corresponding host populations as indicated by significant changes in genotype frequencies both at local (pair-wise comparisons, Table 4 columns 2-4) and global (multiple population comparison, Table 4 last column) levels of comparison and selection coefficients were strongly affected by host genotypes (Table 6). 2) The differences in genetic composition among pathogen populations from different hosts increased over time. Greater differences in genotype frequencies were observed among *P. nodorum *populations sampled from different hosts at late stages of the experiment compared to early stages of the experiment (Tables 3 and 5).

*Phaeosphaeria nodorum *requires 2-3 weeks to complete a cycle of asexual reproduction and the discharge of its pycnidiospores requires rain [45,52-54]. The time intervals between the first collection and second collection were 28 days in the 2003-2004 experiment and 60 days in the 2004-2005 experiment, respectively. Using the meteorological data provided by the local weather station, we estimate that only one generation of asexual reproduction occurred between the first collection and the second collection in 2004 while two asexual generations occurred between the first collection and the second collection in 2005. The significant changes in population composition observed in our experiments indicate strong competition among pathogen genotypes and rapid adaptation to particular hosts, consistent with the hypothesis of rapid pathogen evolution in agricultural ecosystems. Elevated rates of pathogen evolution in agriculture have also been supported empirically for other plant-pathogen interactions. In *Mycosphaerella graminicola*, pathogen populations were collected three times during a single growing season from a susceptible host and rapid directional increases/decreases in genotype frequency were observed for all marked isolates across all replicates [32]. Sequence analyses of plant cell wall degrading enzymes in *M. graminicola *[6] provide further evidence that the evolution of pathogens is accelerated in agricultural ecosystems. Montarry et al. [11] also detected a rapid change of population composition in the potato pathogen *Phytophthora infestans*.

The only strain carrying *ToxA*, SN99CH3.20a, began at a relatively high frequency (17-27%) in each host treatment but was always present at a lower frequency (0-13%) by the final 2005B sample, with an average decrease in frequency of 17%. By comparison, the two other strains (SNCH3.08a and SNCH3.10a) that had an overall decrease in frequency on each host treatment between the first and the final collections showed a decrease averaging less than 2%. A fitness cost associated with *ToxA *was proposed earlier to explain the observed differences in frequencies of *ToxA *positive strains among geographical *P. nodorum *populations [55]. However, this experiment was not designed to determine whether there is a fitness cost associated with carrying *ToxA *and it is not clear whether any of the Swiss wheat cultivars used in this experiment carry the corresponding toxin sensitivity allele *Tsn1*. Therefore, we cannot conclude that the decrease in frequency of SN99CH3.20a reflected a fitness cost associated with *ToxA*.

### The effect of host diversity and partial resistance on the evolution of *P. nodorum*

Both theoretical and empirical studies have demonstrated that increasing genetic diversity in host populations through deployment of cultivar mixtures offers a promising approach to control plant diseases, with the advantages of lower input costs and a reduction in ecological damage compared to use of fungicides while also providing greater yield stability [12,22,56-58]. Investigations of the effect of cultivar mixtures on the evolution of pathogens have been mainly theoretical [12,33,34]. It was hypothesized that increasing genetic diversity in host populations would retard the rate at which pathogens evolve [14,15,21,59] because heterogeneity in the host population would lead to divergent selection pressure on the pathogen population [17]. Several theoretical investigations support the hypothesis that increasing genetic diversity in host populations by using cultivar mixtures will delay the emergence of virulence against major resistance genes [22,33,34]. For quantitative resistance, mixing two cultivars in any proportions may reduce the final virulence attained by the pathogen population and prolong the time needed to reach the equilibrium point of highest virulence [12].

We determined the effect of host diversity on the population dynamics of *P. nodorum *by directly monitoring changes in frequencies of marked strains in a replicated field experiment. Our results support the hypothesis that increasing genetic diversity in host populations through deployment of cultivar mixtures can slow down the rate of evolution in pathogen populations. Multi-population comparisons indicated that the genetic structure of *P. nodorum *populations sampled from the host mixture did not change significantly over two years (Table 4 last column) and displayed the lowest variation in selection coefficients (Table 6). Although this experiment included only one host mixture, a similar evolutionary pattern was observed in field experiments involving other plant pathogens including *Mycosphaerella graminicola *on wheat [32] and *Rhynchosporium secalis *and *Blumeria hordei *on barley [38,60].

It was postulated that partial resistance would retard the evolution of pathogens and thus increase the durability of resistance [24,61]. A theoretical study indicated that virulence of pathogens would evolve slowly in the presence of partially resistant hosts [9]. We tested this hypothesis in our experiments by comparing the changes in frequency of marked pathogen strains competing on susceptible and partially resistant cultivars. In a similar experiment conducted using the wheat-*Mycosphaerella graminicola *pathosystem, we found that the pathogen populations sampled from a partially resistant cultivar exhibited less change in genetic structure over time and smaller selection coefficients than those from a susceptible cultivar [32], consistent with the theoretical expectation. Results from this experiment also support the hypothesis. We detected less significant changes in *P. nodorum *populations sampled from the partially resistant cultivars Tamaro and Runal than from the susceptible cultivar Tirone and resistant cultivar Levis (Table 4). Multi-population comparisons revealed that the changes in genetic structure of the *P. nodorum *populations sampled from the two partially resistant cultivars were significant at the 5% level over the two-years of the experiment, while the differences were significant at the 1% level on the resistant and susceptible cultivars. This result suggests that directional selection also occurs in pathogen populations infecting partially resistant cultivars albeit at a slower pace, leading to the erosion of resistance [2]. The adaptation to partial resistance was also observed in field experiments with the barley scald pathogen *Rhynchosporium secalis *[38] and the potato late blight pathogen *Phytophthora infestans *[19]. Some theoretical analyses of host-pathogen co-evolution suggest that hosts with partial resistance can select for increased virulence in pathogen populations [8]. Our findings agree with these predictions.

### Differential selection on *P. nodorum *strains during parasitic and saprophytic phases

It is hypothesized that saprophytic and parasitic phases of pathogen life cycles may select for different pathogen traits. During the parasitic phase, pathogen strains with a high capacity to exploit the host may have a selective advantage if they are able to produce greater numbers of viable offspring compared to strains with a lower capacity. But the traits that are favored during the parasitic phase of the life cycle may be selected against during saprophytic phases of the life cycle when living hosts are not available [7,23]. The trade-offs that occur between parasitic and saprophytic phases of the life cycle may prevent or delay the emergence of high levels of pathogen virulence.

We found some evidence for differential selection between parasitic and saprophytic phases of the life cycle in the *P. nodorum *populations sampled from Runal, suggesting a fitness cost associated with high virulence during the saprophytic phase [10,62]. On Runal, a significant difference in *P. nodorum *genetic structure was found between 2004A and 2004B but not between 2004A and 2005A, suggesting that selection occurring during the parasitic phase might be offset by selection that occurred during the saprophytic phase. The frequency distribution of isolate SN99CH2.04 also suggested that differential selection might occur between parasitic and saprophytic phases of the pathogen life cycle. This isolate increased in frequency on four of the five treatments (the mixture was the exception) during the parasitic phase of the disease cycle but decreased in frequency during the saprophytic phase on all treatments except the cultivar mixture (Figure 3). This finding indicates that this strain may exhibit higher relative fitness during the parasitic phase on the majority of living host tissue but lower competitive ability during the saprophytic phase on the dead host tissue. Using the same experimental approach, Abang et al. [38] also found that some isolates increased in frequency during the parasitic phase but decreased in frequency during the saprophytic phase in the barley pathogen *Rhynchosporium secalis*.

The lack of evidence for differential selection between parasitic and saprophytic phases in other hosts and isolates may be partially attributed to our sampling strategy. The 2005A collection was made from infected plants several months after the application of the wheat stubble inoculum. Thus at least one cycle of parasitic competition had likely occurred among the pathogen strains before this collection was made. If there was differential selection between the parasitic and saprophytic phases, selection for traits involved in establishment and reproduction during the initiation of the 2005A epidemics may have partially offset selection for traits involved in saprophytic competition. Further experiments with an additional population sample drawn at the beginning of the second cycle of the parasitic phase will be necessary to confirm this hypothesis

Only isolates derived from asexual reproduction of the nine inoculants were used to calculate selection coefficients and determine the effects of host genotypes on the population genetic structure of *P. nodorum*. Isolates having genotypes different from the nine inoculated strains (called novel isolates) were excluded. Because the contribution of mutation to the formation of new genotypes is expected to be trivial within the time scale of this experiment, we believe these novel isolates originated either via immigration from outside of the experimental plots or by recombination among inoculants and/or immigrants within the experimental plots (details in 39). The current paper focuses on the influence of host genotypes and diversity on clonal competition and we believe that excluding these novel isolates did not affect our interpretations.

We used both pair-wise and multiple population comparisons to evaluate the effects of evolutionary time (different sampling points) and host genotypes on the population dynamics of *P. nodorum*. In the multiple population comparisons, pathogen populations from different sampling points or hosts were considered simultaneously in a single analysis. This approach is useful to determine the overall pattern of evolutionary change in pathogen populations over hosts (e.g. last column in Table 4) but cannot be used to determine the sequential change in population structure over time within a host, for example whether the population genetic structure between the first (2004A) and the second (2004B) collection differs more than that between the first and the last (2005B) collection. For the latter case, we adopted pair-wise comparisons that included a Bonferroni correction. We believe that combining these approaches was necessary to achieve a comprehensive analysis of the data.

## Conclusions

Understanding the evolutionary response of pathogen populations to host diversity and environmental changes (such as over-wintering or over-summering between growing seasons) is important for disease management. Many studies on host-pathogen interactions have focused on the development of mathematical models [12,33,63] to predict pathogen evolution in response to different strategies of resistance gene deployment [13,61,64-66]. Very few empirical studies have been conducted to test these theoretical models in agricultural ecosystems. Here, we present empirical evidence that strong selection occurs during both parasitic and saprophytic phases of the disease cycle. Evolution during the parasitic phase occurred most slowly on the cultivar mixture. The same result was also reported in similar experiments conducted with the wheat pathogen *Mycosphaerella graminicola *[32] and the barley pathogen *Rhynchosporium secalis *[38], suggesting that the observed pattern of evolution may be applicable for other splash-dispersed pathogens on cereals.

## Methods

### Experimental design

A two-year mark-release-recapture experiment was conducted at the Agroscope Changins-Wädenswil research center in Changins, Switzerland during the 2003-2004 winter wheat growing season on field allotment-34-North and the 2004-2005 winter wheat growing season on field allotment-35-North. Both fields were grown with a permanent meadow for at least three years prior to the mark-release-recapture experiment. Four commercial Swiss wheat cultivars, namely Levis, Runal, Tamaro and Tirone, were used in these experiments. The varieties differed in quantitative resistance to *P. nodorum *leaf infection according to disease assessments conducted at Changins between 2001 and 2002 [67]. Cultivars Levis, Tamaro and Runal are partially resistant to *P. nodorum *leaf blotch with their levels of resistance decreasing in that order. Cultivar Tirone is susceptible to leaf blotch. Cultivar Levis is partially resistant on the leaves but not on glumes. The four cultivars and a 1:1 mixture of cultivars Runal and Tamaro (5 host treatments in total) were planted in a randomized complete block design (RCBD) with three replications. The field plots were 1.5 m in width and 4.5 m in length. Each wheat plot was surrounded by four equal-sized plots planted with the highly resistant triticale variety Tridel. The experiment was planted on 5 October 2003 in the first year and on 17 October 2004 in the second year using commercial seeds treated with the fungicide Coral (2.38% difenoconazole and 2.38% fludioxonil, 2 ml/kg seeds).

Nine *P. nodorum *isolates collected in 1999 from naturally infected wheat fields near Bern, Switzerland were chosen as inoculants for the 2003-2004 experiment. Each of the isolates had distinct multi-locus haplotypes when assayed with seven single-locus RFLP markers [48], ten polymorphic EST-derived microsatellite markers and one minisatellite marker [68]. Only one of the nine isolates (SN99CH3.20a) carried the *ToxA *gene [55] that encodes a host specific toxin. After completing the field experiments it was discovered that one isolate (SN99CH3.19a) had been replaced by a contaminant of unknown origin, hereafter called C1. Following removal from long-term storage at -80°C, the isolates were first grown on Yeast Maltose Agar (YMA, yeast 4 gl^-1^, maltose 4 gl^-1^, sucrose 4 gl^-1^, agar 10 gl^-1^) at 21°C for ten days and then transferred to 1000 ml flasks containing 300 g of sterilized wheat kernels (cultivar Arina) in a dark incubator at 4°C. Three months later the infected wheat kernels were harvested and ground to a powder using a gristmill. The powdered kernels were mixed with distilled water and the spore suspension was filtered through cheese cloth and glass-wool. The spore suspension from each isolate was adjusted to 10^6 ^spores per ml using a hemacytometer and mixed in equal proportions. A surfactant (Tween 20) was added to the spore suspension at the rate of one drop per 50 ml. The aqueous spore suspension was applied onto disease-free wheat seedlings at growth stage 31 on 11 May 2004. Each field plot was sprayed with 500 ml of the calibrated spore suspension. To maximize the humidity and increase the probability of infection, inoculations were carried out in the late afternoon on a cloudy day and the inoculated seedlings were covered with a plastic tarp for 24 hours.

The source of primary inoculum in the 2004-2005 experiment was the infected straw and other plant debris saved from the first year's experiment. After harvesting the grain at the end of July 2004, the straw and other plant debris in each plot were collected and stored separately in burlap potato sacks for 3 months. The sacks were stored in a dry, dark and cool room to allow the development of the saprophytic phase without the risk of excessive moulding. At the beginning of tillering (Zadoks stage 13 to 21, 69), the straw was applied onto the corresponding host plots.

A total of four fungal collections were made across the two growing seasons. The first collection, hereafter called 2004A, was made on 4 June 2004 from the third or fourth full leaf [60] at three weeks after the artificial inoculation. The second collection, hereafter called 2004B, was made on 2 July 2004 from flag leaves. The third collection, hereafter called 2005A, was made on 11 April 2005 from the second true leaf and the last collection, hereafter called 2005B, was made on 10 June 2005 from the third true leaf. For each collection, 30 to 40 leaves were collected from each inoculated plot at intervals of approximately 20 cm along transects within the inner rows of the field plots. In most cases, only one isolate was made from each infected leaf. Because many lesions did not contain pycnidia, the total number of isolations made was much lower than the number of wheat leaves collected. For some collections with very low levels of infection, two isolations were made from the same leaf. In these cases, isolations were made from clearly separated lesions to minimize the probability of sampling isolates from the same infection. Our earlier work showed that *P. nodorum *isolations made from different lesions within an infected leaf usually contain different genotypes, suggesting they originate from different infection events [70].

### DNA extraction and microsatellite data collection

DNA was extracted from each isolate using the DNeasy Plant Mini DNA extraction kit (Qiagen GmbH, Germany) according to the specifications of the manufacturer. The genotype of each isolate was determined using the same ten EST-derived microsatellite markers (SN1, SN3, SN5, SN11, SN15, SN16, SN17, SN21, SN22, and SN23) and one minisatellite marker (SN8) used to mark the nine inoculants. Multiplexed polymerase chain reactions (PCR) were carried out with fluorescently labeled primers using the same conditions described previously [68]. The PCR products were first cooled on ice for 2 min and then separated on an ABI PRISM 3100 sequencer according to the manufacturer's instructions (Applied Biosystems). Fragment sizes were estimated and alleles were assigned using the program GENESCAN 3.7 (Applied Biosystems).

### Data analysis

The multilocus haplotype (MLHT) for each isolate was formed by joining the alleles at each of the 11 marker loci. Isolates with the same MLHT were considered to be clones, the products of asexual reproduction for a particular genotype. Because fungal collections were found to consist of both inoculated and novel isolates (novel isolates are defined as isolates having MLHTs different from any of the nine inoculated isolates, 39), only isolates derived from asexual reproduction of the nine inoculants were used to calculate selection coefficients and determine the effects of host genotypes on the population genetic structure of *P. nodorum*. The novel isolates, which originated either via immigration from outside of the experimental fields or by recombination among inoculated isolates and immigrants, were considered in a separate publication [39].

Pair-wise and multiple population comparisons in genotype frequencies were performed using contingency χ^2 ^tests to detect differences in pathogen populations sampled from different hosts or sampling times [71]. Pair-wise comparisons were corrected using a sequential Bonferroni procedure as described previously [72]. Multiple population comparisons were conducted by using all populations sampled from the same host across different sampling points simultaneously, generating a *c x l *contingency table, where *c *is the number of sampling points and *l *is the number of genotypes detected. The association between sampling time and differences in genotype frequency among populations was evaluated using simple linear correlation. In this analysis, differences in genotype frequency among populations were measured by G_ST _[73,74]

Selection coefficients of the inoculated isolates within each host treatment were estimated simultaneously by setting the coefficient of the most-fit isolate (the isolate with the greatest increase in frequency over the considered time period) to zero as described previously [32]. Let the initial frequency for genotype *G_1_, G_2 _*...... and *G_i _*be , , ..... and  and their selection coefficients per generation be *s_1_*, *s_2_*, ...... and *s_i_*, respectively. Then the average fitness for this population at generation t = 0 will be:(1)

And the frequency of genotypes *G_1_, G_2_*...... and *G_i _*after one generation of selection will be:(2)

And the frequency for genotype *G_1_, G_2_*, ...... and *G_i _*after t generations of selection will be:(3)

Let G_j _(i ≠ j) be the most-fit genotype, i.e. s_j _= 0, then the average fitness () in the t-1 generation will be:(4)

S_1_, s_2 _...... s_i _can be obtained by substituting equation 3 with equation 4, leading to the general solution of:(5)

This estimate of selection coefficient measures the overall fitness of a genotype relative to the most-fit isolate in a population during the entire life cycle of the pathogen, taking into account its ability to infect, colonize, reproduce and spread [32]. For example, a genotype with a selection coefficient of 0.30 has 30% lower fitness than the most-fit genotype. Selection coefficient is different from selection intensity, a term used mainly in breeding and quantitative genetics to quantify a potential evolutionary gain after selecting a pool of parents from a variable population. To make more robust estimates, selection coefficients were estimated for each host treatment by pooling together data from different replications using only five of the nine inoculants for the 2004A and 2004B collections. Isolate SN99CH3.23a was not recaptured during the course of the experiment and the other three inoculants were recovered at frequencies too low to make meaningful estimates of selection coefficients. Selection coefficients were not estimated for the 2005 collections due to small sample sizes (9-18 isolates/host treatment) remaining after the novel isolates were excluded from 2005B. The means and standard deviations of the selection coefficients were generated based on 100 resamples of the original genotype frequencies using the Excel add-in PopTools 2.7 (CSIRO, Australia). Tukey's significant differences implemented in SYSTAT were used to compare selection coefficients among the five inoculants.

## Authors' contributions

RJS inoculated field plots, isolated fungal strains, conducted molecular assays, contributed to analysis of data and manuscript preparation; BAM contributed to the experimental design and coordination of the project, interpretation of the results and manuscript preparation (including Figures 1 and 2): FM prepared pathogen inoculum, carried out field experiments and participated in sample collections; JZ conceived the study, contributed to the experimental design and coordination of the project, interpretation of the results and led the statistical analysis of data and manuscript preparation. All authors read and approved the final manuscript.
